# Correction: Mohd Azmi et al. Application of Plant Proteases in Meat Tenderization: Recent Trends and Future Prospects. *Foods* 2023, *12*, 1336

**DOI:** 10.3390/foods13030379

**Published:** 2024-01-24

**Authors:** Syahira Izyana Mohd Azmi, Pavan Kumar, Neelesh Sharma, Awis Qurni Sazili, Sung-Jin Lee, Mohammad Rashedi Ismail-Fitry

**Affiliations:** 1 Department of Food Technology, Faculty of Food Science and Technology, Universiti Putra Malaysia (UPM), Serdang 43400, Selangor, Malaysia; izyanaazmi@gmail.com; 2 Department of Livestock Products Technology, College of Veterinary Science, Guru Angad Dev Veterinary and Animal Sciences University, Ludhiana 141004, Punjab, India; pavankumar@gadvasu.in; 3 Institute of Tropical Agriculture and Food Security, Universiti Putra Malaysia (UPM), Serdang 43400, Selangor, Malaysia; 4 Division of Veterinary Medicine, Faculty of Veterinary Sciences and Animal Husbandry, Sher-e-Kashmir University of Agricultural Sciences and Technology of Jammu, Ranbir Singh Pura 181012, Union Territory of Jammu and Kashmir, India; drneelesh_sharma@yahoo.co.in; 5 Department of Animal Science, Faculty of Agriculture, Universiti Putra Malaysia (UPM), Serdang 43400, Selangor, Malaysia; awis@upm.edu.my; 6 Halal Products Research Institute, Universiti Putra Malaysia (UPM), Serdang 43400, Selangor, Malaysia; 7 Department of Applied Animal Science, College of Animal Life Sciences, Kangwon National University, Chuncheon 24341, Republic of Korea

## Missing Citation

In the original publication [1], “Fan et al. [98]” [2] were not cited. The citation has now been inserted in “5. Novel Technological Interventions”, “Paragraph 1” and should read:

“Figure 3. Various prospects of the application of novel processing technologies in the tenderization of meat via the application of plant proteases. [Source of underwater shockwaves—Fan et al. [98]]”.

The authors state that the scientific conclusions are unaffected. This correction was approved by the Academic Editor. The original publication has also been updated.
